# The Relationship between Obstructive Sleep Apnea and Atrial Fibrillation: A Complex Interplay

**DOI:** 10.1155/2013/621736

**Published:** 2013-02-26

**Authors:** Jacqueline M. Latina, N. A. Mark Estes, Ann C. Garlitski

**Affiliations:** Department of Medicine, New England Cardiac Arrhythmia Center, The Tufts Cardiovascular Center, Tufts University School of Medicine, 750 Washington Street, Boston, MA 02111, USA

## Abstract

In recent years, growing evidence suggests an association between obstructive sleep apnea (OSA), a common sleep breathing disorder which is increasing in prevalence as the obesity epidemic surges, and atrial fibrillation (AF), the most common cardiac arrhythmia. AF is a costly public health problem increasing a patient's risk of stroke, heart failure, and all-cause mortality. It remains unclear whether the association is based on mutual risk factors, such as obesity and hypertension, or whether OSA is an independent risk factor and causative in nature. This paper explores the pathophysiology of OSA which may predispose to AF, clinical implications of stroke risk in this cohort who display overlapping disease processes, and targeted treatment strategies such as continuous positive airway pressure and AF ablation.

## 1. Introduction

Obstructive sleep apnea, a common breathing disorder, is characterized by recurrent episodes of airway collapse resulting in occlusion of airflow during sleep. These episodes of hypopnea and apnea can manifest as transient or prolonged hypoxemia, sleep arousals, and sympathetic nervous system activation, resulting in symptoms such as snoring, headaches, daytime sleepiness, and impaired alertness [1]. Approximately 3–7% percent of the adult population in the United States is affected by OSA, and that number is likely an underestimate as it goes undiagnosed in many cases [2–4]. Another condition which is also prevalent as well as undiagnosed in many circumstances is atrial fibrillation (AF). It is predicted that by 2050, more than 10 million Americans will have AF and possibly up to 16 million if the increase in incidence continues on the same trajectory [5]. The question has been posed, on more than one occasion, how these two common entities of OSA and AF impact one another. Although the relationship between sleep-disordered breathing and arrhythmias was proposed a few decades ago [6], only recently has it been recognized that OSA seems to be correlated with AF (see Table 1) [7–15].

The studies in Table 1 suggest the increased risk of AF in OSA patients, but the quality of the data is limited [7–15]. Known risk factors for AF include age, male gender, smoking, obesity, hypertension, diabetes mellitus, myocardial infarction, congestive heart failure, and cardiac surgery [15]. Of the six studies evaluated in Table 1, only two adjusted for male gender [8, 11], one for hypertension [9], and none controlled for diabetes mellitus, or heart failure [8, 16]. In the Sleep Heart Health Study, Mehra et al. demonstrated that the risk of AF among patients with OSA is about four times than of those with no sleep-disordered breathing (adjusted OR 4.02; 95% CI 1.03–15.74) [8].

It is important to note that the MrOS Study and the Sleep Heart Health Study compared the incidence of nocturnal arrhythmias in subjects with and without sleep-disordered breathing, suggesting that sleep disordered breathing directly affects heart rhythm [8, 10]. Furthermore, Gami et al. showed that for patients with OSA under age 65, the hazard ratio of developing any type of AF over the course of approximately 5 years was 3.29 (95% CI 1.35–8.04) [11]. These studies suggest that OSA predisposes patients to develop AF, but it is not clear whether the relationship is independent of hypertension, diabetes mellitus, or other confounding factors. A study by Mehra et al. in 2009 (MrOS Study) demonstrated that AF was associated with central sleep apnea but not obstructive sleep apnea after adjusting for confounding factors [15]. A prospective randomized trial is needed to confirm the association between OSA and AF.

Conversely, it has been shown that patients with AF appear to be more likely to have OSA compared to the general population [7, 12, 13]. In a landmark study in 2004, Gami et al. showed that after adjusting for body mass index, neck circumference, hypertension, and diabetes mellitus, approximately half of patients with AF were likely to have OSA (adjusted OR 2.19, 95% CI 1.40–3.42, *P* = 0.0006) [7]. However, conflicting data does exist, as one study did not show any difference in the prevalence of OSA among patients with AF compared to gender-, age-, and cardiovascular morbidity-matched community controls [17].

The question remains whether the association of OSA and AF is based on mutual risk factors, such as obesity and hypertension, or whether OSA independently, directly, and definitively leads to AF. This paper will delineate the underlying pathophysiologic mechanisms that support the plausibility of this interplay. In addition, we will describe the clinical implications of stroke risk and discuss treatment strategies such as continuous positive airway pressure (CPAP) and AF ablation.

## 2. Methods

This is a comprehensive review based on a literature search of the Ovid Medline database (1946 to September 2012) and Cochrane Database of Systematic Reviews. The following searches were conducted: (1) “sleep apnea” or “obstructive sleep apnea” AND “atrial fibrillation” (2) “sleep apnea” AND “catheter ablation” (3) “sleep apnea” AND “pulmonary vein isolation” (4) “CPAP” AND “atrial fibrillation” and (5) “obstructive sleep apnea” AND “stroke.” The paper intends to address the underlying pathophysiology of obstructive sleep apnea leading to atrial fibrillation. We will discuss the impact of OSA treatment on AF and the correlation between OSA and stroke.

## 3. Pathophysiology of Obstructive Sleep Apnea

The upper airspace in the pharyngeal region is a combination of muscles and soft tissue, differing from the cartilaginous trachea and bronchi in that the airspace can collapse [18]. Sleep naturally causes a loss of tone in the muscles surrounding the pharynx, reducing the pharyngeal airspace and increasing resistance to airflow [18]. Obesity is the main risk factor for obstructive sleep apnea, as the fat accumulates and narrows the lumen of the pharynx [19]. In individuals with narrowed and compliant airways at baseline, the reduction of muscle tone leads to occlusion of the pharynx, resulting in hypopneas, complete obstruction of airflow, hypoxia, and hypercapnia. Unsuccessful inspiratory efforts against an occluded airway lead to a precipitous drop in intrathoracic pressure with a subsequent increase in afterload [20], enhancement of venous return, leading to distention of the right ventricle and shifting of the interventricular septum. The combination of interventricular septal shift, impeding left ventricular filling, and an increased afterload leads to a decrease in left ventricular stroke volume and thus a temporary fall in cardiac output [19].

### 3.1. Sympathetic Activation

During apneic episodes, the sympathetic nervous system is activated by pulmonary stretch receptors normally quiescent during sleep [19]. Hypoxia and hypercapnia increase sympathetic nervous system activation by stimulating peripheral and central chemoreceptors [21, 22]. The resulting sympathetic stimulation causes vasoconstriction and results in a significant increase in blood pressure [21]. Immediately after the apneic period resolves, there is a significant elevation in heart rate and blood pressure [19]. These surges can occur hundreds of times per night in someone with severe OSA, leading to large variability in heart rate and blood pressure. Sleep apnea has been strongly correlated with hypertension in several studies [23–26]. Nevertheless, more recent studies suggest that the relationship between OSA and hypertension may not be causal and may be confounded by obesity [27, 28]. Normotensive patients newly diagnosed with OSA have faster resting heart rates [29], increased blood pressure variability [30] and are predisposed to developing hypertension [31] and end-organ damage [32].

### 3.2. Systemic Vascular Responses and Inflammation

Inflammatory mediators such as cytokines and adhesion molecules appear to be increased in OSA [33, 34]. C-reactive protein (CRP), a marker of systemic inflammation, is also increased in OSA [35, 36]. Oxidative stress on neutrophils and monocytes results in higher levels of oxygen radicals in patients with OSA [37]. Patients with OSA have higher plasma CRP concentrations that increased corresponding to the severity of their apnea-hypopnea index score [35]. CRP and interleukin-6 (IL-6) levels were significantly higher in patients with OSA compared to obese control subjects (CRP *P* < 0.001, IL-6 *P* < 0.05) [36]. In turn, Yokoe et al. also showed that CPAP significantly alleviated the effect of OSA on CRP and IL-6 levels [36]. Treatment of OSA with CPAP downregulates the expression of adhesion molecules and decreases the formation of reactive oxygen species [34, 37].

## 4. Diagnosis of Obstructive Sleep Apnea

The gold standard of diagnosis is a polysomnographic study which records sleep and breathing in a sleep laboratory overnight. A hypopneic episode is defined to fit one of the following criteria: greater than 50% reduction in airflow or tidal volume for at least 10 seconds, moderate reduction in airflow (less than 50%) with arterial oxygen desaturation greater than 3%, or moderate reduction in airflow with electroencephalographic evidence of arousal from sleep [38]. Arousal from sleep is defined as transient awakening for less than 10 seconds [19]. The severity of OSA is measured by the apnea-hypopnea index (AHI), the frequency of apneas and hypopneas per hour of sleep. An apnea-hypopnea index ≥ 5 is considered mild, while AHI ≥ 15 is moderate-to-severe OSA [39, 40].

Albuquerque et al. recently showed that daytime sleepiness symptoms did not correlate with the presence of sleep-disordered breathing in patients with AF [41]. Patients may be asymptomatic and thus unaware of their sleep-disordered breathing, raising the question of not only how to manage these patients but also how to screen for them. 

### 4.1. Risk Factors

Risk factors for developing OSA include obesity, neck circumference, male gender, increasing age, alcohol use, smoking, menopausal status, and black race [38, 42]. Obesity is the most important risk factor, as approximately 70% of patients with OSA are obese [38]. As obesity is at epidemic proportions, the number of people affected by OSA is likely to increase accordingly. The relationship between OSA and AF is complicated by the fact that obesity is also an independent predictor of AF. Furthermore, in patients with OSA who are under 50 years of age, there has been evidence to show that they are more likely to suffer hypertension, AF, and all-cause mortality [11, 43, 44].

## 5. Pathophysiologic Mechanisms of Obstructive Sleep Apnea Influencing Atrial Fibrillation

Multiple pathophysiologic mechanisms have been proposed to explain the association between OSA and AF, and it is likely that the interplay among them, rather than one factor, predisposes OSA patients to AF (Table 2). Data suggests that OSA may induce cardiac remodeling [45–48], increase sympathetic activity [21, 49, 50], and cause systemic inflammation [33–36].

### 5.1. Structural and Functional

Recent studies have examined atrial remodeling of structural and electrical components in patients with OSA to account for predisposition to AF [45–48, 51–54]. Sudden negative intrathoracic pressures may lead to repetitive atrial stretch and gradually lead to left atrial enlargement [55]. As a result of left atrial enlargement, there may be remodeling at the pulmonary vein ostia, a site known to initiate and propagate AF [56]. Patients with severe OSA (AHI > 15) undergoing catheter ablation had significantly larger left atria indexed to body surface area compared to those patients those with an AHI < 15 (*P* = 0.009) [46], confirming the results found in other studies [55, 57]. Since an enlarged left atrium is a risk factor for AF [58], a plausible mechanism for OSA leading to a predisposition to AF is structural remodeling.

Severe OSA patients were also shown to have extensive areas of low voltage or electrical silence, and conduction abnormalities as indicated by prolonged P-wave durations, slower atrial conduction velocity and sinus node recovery times [46]. A study of Japanese males with OSA found that the atrial electromechanical activation time (EMAT) was significantly longer than control subjects [59]. Yagmur et al. showed that interatrial and intra-atrial electromechanical delay were significantly prolonged in patients with moderate-to-severe OSA compared to controls (*P* < 0.0001) [60]. These electrical abnormalities may underlie a predisposition of OSA patients to AF.

In addition to causing left atrial enlargement and atrial conduction abnormalities, OSA also appears to induce left ventricular hypertrophy (LVH). An observational study of 51 males with OSA showed that LVH and right ventricular hypertrophy were more prevalent in men with severe OSA compared to those with mild OSA [61]. All the patients with LVH had hypertension. Several studies have established that OSA is significantly associated with hypertension, especially in patients younger than 65 years [23–25, 43, 57, 62, 63]. However, there is conflicting data from the Sleep Heart Study that suggests this association was accounted for by obesity and not statistically significant after correcting for body mass index [28].

Drager et al. looked at arterial stiffness by measuring pulse wave velocity and heart structure in OSA patients with and without hypertension [57]. They demonstrated that patients with untreated OSA had increased left atrial diameter, septal wall thickness, left ventricular (LV) posterior wall thickness, LV mass index, and LVH, even controlling for cardiovascular risk factors [57]. These indicators showed additive effects of hypertension and OSA. 

These studies support the concept that OSA leads to cardiac remodeling in order to compensate for the repeated stress of an occluded airway. On the contrary, other studies did not show any difference in LV mass between obese patients with and without OSA [52, 55, 64].

The Müller maneuver, occluded inspiration, can generate negative intrathoracic pressure of −60 to −80 mm Hg [65], to simulate OSA [66, 67]. Orban et al. showed that when healthy individuals performed the Müller maneuver without confounding hypoxia and comorbidities, there was a decrease in LV ejection fraction and left atrial volume [66]. Immediately after release of the maneuver, there was a compensatory increase in cardiac output above baseline. Koshino et al. showed that during the Müller maneuver ventricular longitudinal deformation, as measured by strain and strain rate, was significantly reduced [67]. These variations in left atrial size and cardiac output as well as impairment of ventricular mechanics may result in long-term compensation, potentially increasing the risk of AF and heart failure in OSA patients.

There are a number of animal models that further detail these structural changes. In a pig model, tracheal occlusions increased AF susceptibility [68]. A recent study evaluating AF inducibility in rats showed that the likelihood of AF induction in obese rats was greater than in lean rats (85.7% versus 27.8%, *P* < 0.001) [69]. Their animal model suggests that obesity and OSA interrelate to promote AF by promoting left atrial dilation and LV dysfunction.

Studies have suggested that OSA is associated with diastolic dysfunction [55, 70, 71]. Fung et al. showed a correlation between the severity of OSA and the degree of left ventricular diastolic dysfunction in an uncontrolled study [71]. Kraiczi et al. showed that nocturnal oxygen desaturations were found to be associated with left ventricular diastolic dysfunction [70]. Otto et al. confirmed these findings, showing that 82% of patients with OSA had indicators of early diastolic dysfunction compared to 26% of obese control patients without OSA (*P* = 0.001) [55]. Cardiac structural and functional changes leading to reduced diastolic function may predispose OSA patients to AF.

### 5.2. Hypoxemia

Hypoxemia and hypercapnia affect sympathetic nerve activity, causing vasoconstriction and, as a result, hypertension [21] which, in turn, is a risk factor for AF. Studies suggest that the severity of OSA as measured by nocturnal oxygen desaturations correlates to the prevalence of AF [9, 11]. In a study of Japanese men, Tanigawa et al. looked at 3% oxygen desaturation indices to measure apneic events during sleep [9]. Patients with more desaturation events had a trend toward an increased risk of AF; the multivariate adjusted OR was 2.47 (95% CI 0.91–6.69) and 5.66 (95% CI 1.74–18.34), for mild and severe OSA, respectively (*P* = 0.02) [9]. In a retrospective cohort study, mean followup of 4.7 years, risk factors for AF in individuals ≤65 years included male gender (HR 2.66, 95% CI 1.33–5.30), age (HR 2.04, 95% CI 1.48–2.80), coronary artery disease (HR 2.66, 95% CI 1.46–4.83), body mass index (HR 1.07, 95% CI 1.05–1.10), and nocturnal oxygen saturation (HR 3.29, 95% CI 1.35–8.04) [11]. Of interest, the magnitude of nocturnal oxygen desaturation was a risk factor for AF, independent of a diagnosis of OSA. The study by Gami et al. was novel in that it demonstrated the degree of nocturnal desaturations predicts AF within approximately 5 years of diagnosis of OSA [11]. Their findings implicate hypoxemia as a potential important mediator of AF. 

In a prospective study, patients with greater falls in nocturnal oxygen saturation had a higher recurrence rate of AF after cardioversion [72]. In the untreated OSA patients, the AF recurrence rate exceeded 80%, compared to 42% in the treated OSA group and 53% in the control patients [72]. The higher recurrence rate among controls compared to treated OSA patients suggests that some of the controls may have had undiagnosed OSA.

### 5.3. Autonomic Tone

Frequent episodes of desaturation can lead to sympathetic activation and contribute to cyclical increases in blood pressure. The pulmonary vein ostia are innervated by adrenergic and vagal nerves, which have been implicated in the development of AF and targeted by ablation [73]. Noda et al. showed that in patients with OSA, nocturnal plasma norepinephrine levels were correlated with the duration of oxygen desaturation [61].

Animal studies have been performed to attempt to elucidate the autonomic mechanisms that affect OSA and AF. Using a canine model, Ghias et al. demonstrated that they were able to induce AF during acute apnea episodes, and significantly repress AF inducibility after either autonomic blockade or neural ablation of the right pulmonary artery ganglionic plexus [74].

Linz et al. showed that atrial effective refractive period (AERP) was shortened with applied negative tracheal pressure in a swine model, resulting in greater AF inducibility [68]. They further showed that negative tracheal pressure-induced AF inducibility was completely inhibited by atropine and bilateral vagotomy. Based on these results, they concluded that negative tracheal pressure-triggered AF was mediated by vagal activation alone because it was inhibited by atropine or vagotomy. 

Their group also used a swine model to demonstrate that blocking early and late potassium currents, with an early activated potassium current blocker (AVE0118), amiodarone, and sotalol, did not affect the negative tracheal pressure-induced AERP shortening [75]. From this data, they concluded that it is difficult to inhibit arrhythmias caused by negative tracheal pressure (simulating OSA) with class III antiarrhythmic drugs. Nevertheless, they did find that an early and a late activated potassium current in combination could reduce negative tracheal pressure-induced AERP shortening and thus AF inducibility [75].

In a more recent animal study, Linz et al. revealed that renal denervation reduced AF inducibility during applied negative tracheal pressure. Postapneic hypertension was inhibited by renal denervation but not changed by atenolol [79]. Thus, they concluded that renal denervation had antiarrhythmic effects and reduced blood pressure elevations following obstructive apneic episodes. 

The pathophysiologic mechanisms of cardiac remodeling, structural changes such as left atrial dilation and electrical remodeling, hypoxemia, and autonomic dysregulation have been implicated as predisposing factors in OSA patients to AF. Animal studies as well as human data are beginning to illustrate these mechanistic links. 

## 6. Treatment of Atrial Fibrillation in Obstructive Sleep Apnea Patients

Continuous positive airway pressure is the treatment of choice for OSA [80]. Tkacova et al. showed that CPAP reduced LV afterload and heart rate during sleep in patients with OSA and congestive heart failure [81]. Pepperell et al. demonstrated that nasal CPAP treatment lowered blood pressure and improved symptoms of daytime sleepiness and quality of life [26, 82–84]. CPAP also helps alleviate brady-arrhythmias associated with OSA [85]. Although these studies have not shown a direct impact on AF specifically, they influence the mechanisms which may lead to AF.

There have only been a few studies which have examined the impact of CPAP therapy on AF in OSA patients (please see Table 3) [72, 76–78]. None of the four studies were randomized. A Japanese group looked at the effect of CPAP on nocturnal arrhythmias and found that CPAP significantly decreased the occurrence of paroxysmal AF (*P* < 0.001) [77]. As previously noted, Kanagala et al. demonstrated that patients treated appropriately with CPAP had a lower recurrence of AF after cardioversion [72]. Anter et al. and Patel et al. also demonstrated that among patients who underwent pulmonary vein isolation for AF, there was a significant decrease in AF recurrence in CPAP-treated patients compared to untreated patients [76, 78]. While these studies suggest that CPAP may alleviate the obstructive mechanisms leading to AF, a randomized trial looking at the effectiveness of CPAP to decrease the incidence of AF has not been performed. One trial, recently completed in May 2012, aimed to determine the effect of CPAP on the rate of recurrence of AF after cardioversion therapy [99]. There is an ongoing trial currently recruiting participants aimed to determine the incidence of new onset atrial fibrillation among patients with severe OSA using an implantable loop recorder [100]. The study will also examine the effect of CPAP on the incidence of AF and is estimated to complete in 2014. These trials should help determine if CPAP affects the onset of new or recurrent AF.

Given the increased risk of cardiac death at night in OSA patients (RR 2.57, 95% CI 1.87–3.52) [101], and the evidence suggesting that CPAP therapy protects against death secondary to cardiovascular causes in patients with OSA [102], it would be prudent to evaluate the effectiveness of CPAP as it relates to incidence of arrhythmias. If CPAP therapy is found to be effective for arrhythmia prevention, it could be considered as upstream therapy prior to or in conjunction with antiarrhythmic medication, pacemaker placement for bradyarrhythmias, or catheter ablation for AF. Early intervention with CPAP, a noninvasive therapy, is a reasonable consideration as patients with severe OSA are less likely to respond to antiarrhythmic drug therapy [103].

## 7. Catheter Ablation of Atrial Fibrillation in OSA Patients

Although pulmonary vein isolation (PVI) has been established as an effective treatment for AF [56, 104–106], some patients experience conduction recurrence across a previously disconnected pulmonary vein. Sauer et al. characterized this group and concluded that acute return of pulmonary vein conduction is more likely after successful PVI in patients who are elderly, hypertensive, with nonparoxysmal AF, a large left atrium, and sleep apnea [107].

A recent meta-analysis by Ng et al. determined that OSA patients have a 25% greater AF recurrence rate after PVI (RR 1.25, 95% CI 1.08–1.45, *P* = 0.003) [86]. The meta-analysis looked at six studies published between 2008 and 2010 and included 3,995 patients [78, 87–91]. These studies are summarized in Table 4. Subgroup analysis demonstrated that patients diagnosed with OSA using the Berlin Questionnaire (BQ) did not have a greater risk of AF recurrence after catheter ablation, but patients diagnosed using polysomnography were at increased risk. The BQ, a popular diagnostic tool due to its relative ease and cost-effectiveness, may overestimate the number of OSA patients, leading to a greater false-positive rate [89]. According to Jongnarangsin et al., after multivariate analysis, OSA was the strongest predictor of recurrent AF after catheter ablation, associated with a threefold increase in the probability of recurrence (OR = 3.04, 95% CI 1.11–8.32, *P* = 0.03) [91]. A recent study showed that moderate-to-severe sleep-disordered breathing (AHI ≥ 15) was an independent predictor of AF recurrence after PVI with cryoballoon technique (HR 2.95, *P* = 0.04) [108].

As previously noted, treatment with CPAP was shown by Patel et al. to improve PVI success rate [78]. Patients not treated with CPAP had a higher prevalence of nonpulmonary vein triggers, which is likely a reflection of electrical and structural remodeling of the atria, and they were 8 times more likely to fail the procedure [78]. Patients with OSA should be given special consideration when they are being evaluated for catheter-based AF ablation, and OSA treatment should be maximized to improve ablation results.

## 8. OSA and Stroke Risk

Obstructive sleep apnea also increases the risk of stroke and death [94, 95, 109]. It remains unclear if this increased risk of stroke is related to the increased risk of paroxysmal AF, or if this is an independent factor. According to a meta-analysis by Loke et al. of 8345 participants, OSA more than doubled the risk of stroke (OR 2.24, 95% CI 1.57–3.19, *I* ^2^ = 7%), but only three of the five studies recorded the presence of AF [110]. Studies examining the relationship between OSA and stroke risk are summarized in Table 5. 

There are three ongoing trials examining whether CPAP treatment reduces the rate of cardiovascular events and stroke. The RICCADSA Trial at Skaraborg Hospital aims to determine if CPAP reduces the rate of revascularization, MI, stroke, and cardiovascular mortality in 400 patients over a mean of 3 years followup in coronary artery disease patients without daytime sleepiness [111]. There are two other multicenter randomized clinical trials estimated to complete in 2014 and 2015 which also aim to determine if CPAP will reduce cardiovascular death and nonfatal events such as MI, stroke, and heart failure [112, 113]. While these studies are not looking directly at the relationship between stroke and AF, they may shed some light on whether CPAP lowers the risk of stroke in OSA patients. 

The Framingham Study defined AF as a risk factor for stroke [114]. In 2001, the CHADS_2_ score was developed to aid the decision to prophylactically anticoagulate a patient to decrease the risk of stroke [115]. This scoring system awards one point for the risk factors congestive heart failure, hypertension, age ≥ 75, and diabetes mellitus, and two points for previous stroke or transient ischemic attack. A score of two or higher implies the patient should be anti-coagulated with warfarin or another appropriate anticoagulant than aspirin or clopidogrel if there are no contraindications. The CHADS_2_ score was developed before OSA was recognized to increase the risk of stroke, and it has recently been debated whether OSA should be added to the CHADS_2_ score [116]. The relative risks for hypertension, age, diabetes, and prior stroke/TIA are similar to that of OSA [116]. Obstructive sleep apnea is often associated with significant cardiac risk factors such as hypertension, diabetes, and obesity, further increasing the risk of stroke [117]. A large prospective trial is needed to determine if the CHADS_2_ score should be the CHADSS_2_ score, adding one more “s” for sleep apnea.

## 9. Conclusion

In obstructive sleep apnea, left atrial remodeling, diastolic dysfunction, increased autonomic tone, inflammatory mediators, and hypertension are potential factors which coalesce to amplify the risk of AF. OSA also increases the risk of stroke, and it remains unclear whether this is related to paroxysmal AF or an independent risk factor. Patients with OSA and AF are prone to recurrent AF after cardioversion or catheter ablation. CPAP is the treatment of choice for OSA, and it may have some antiarrhythmic effects. However, a prospective randomized control trial is needed to determine the effect of CPAP on AF prevention. General internists and electrophysiologists alike should screen patients for OSA when considering possible interventions for atrial fibrillation. 

## Figures and Tables

**Table 1:** Risk of AF in OSA patients.

Investigator	Number of patients	Methods of diagnosis for OSA	Control group	Risk factor adjustments	Results
Guilleminault et al. (1983) [6]	400	PSG	No	N/A	Cardiac arrhythmias observed in 48% of patients with OSA
Mooe et al. (1996) [14]	121	PSG	Yes	Age.	Risk of AF after CABG in OSA patients, OR 2.8 (1.2–6.8)
Mehra et al. (2006) [8]	566	PSG	Yes	Age, gender, BMI, coronary heart disease.	Risk of AF in OSA patients, adjusted OR 4.02 (1.03–15.74)
Tanigawa et al. (2006) [9]	1763	Pulse oximeter during sleep	Yes	Age, BMI, alcohol intake, blood pressure, hypertension, and antihypertensives.	Risk of AF for severe OSA, adjusted OR 5.66 (1.75–18.34)
Gami et al. (2007) [11]	3542	PSG	Yes	Age, gender, coronary artery disease, BMI.	Incident AF in OSA for patients age <65, HR 3.29 (1.35–8.04)
Monahan et al. (2009) [10]	2816	PSG	Yes	Subjects serve as their own controls during event-free periods.	Risk of AF after a respiratory disturbance compared to normal breathing, OR 17.9 (2.2–144.2)
Mehra et al. (2009) [15]	2911	PSG	Yes	Age, race, BMI, HTN, DM, CAD, pacemaker, cholesterol.	Increasing OSA quartile associated with CVE (*P* = 0.01) but not AF.

PSG: polysomnogram; CABG: coronary artery bypass graft; BMI: body mass index; HTN: hypertension; DM: diabetes mellitus; CAD: cardiac disease; OR: odds ratio; HR: hazard ratio; CVE: complex ventricular ectopy.

**Table 2:** Pathophysiologic mechanisms.

Mechanisms of AF development in obstructive sleep apnea
Negative intrathoracic pressure
Hypoxemia
Hypercapnia
Autonomic nervous system activation
Inflammation
Arterial stiffening
Hypertension
Left ventricular hypertrophy
Diastolic dysfunction
Interventricular septal wall thickening
Left atrial enlargement
Atrial electromechanical remodeling

**Table 3:** Does CPAP improve AF outcomes?

Investigator	Number of patients	Follow-up period (months)	Methods	Results
Anter et al. (2012) [76]	426	26 ± 18	Prospective consecutive patients	After PVI, 72% of the CPAP patients and 62% of the non-CPAP patients were free of AF (*P* = 0.005)
Abe et al. (2010) [77]	1394	N/A	Prospective trial	CPAP significantly reduced the occurrences of paroxysmal AF (*P* < 0.001)
Patel et al. (2010) [78]	640 OSA patients, 2360 controls	32 ± 14	Prospective consecutive patients	After PVI, 79% of CPAP users compared to 68% of non-CPAP users were free of AF (*P* = 0.003)
Kanagala et al. (2003) [72]	39 OSA patients, 79 controls	12	Prospective trial	Recurrence on CPAP was 42% versus 82% in non-CPAP group (*P* = 0.013); and 53% in controls (*P* = 0.009)

CPAP: continuous positive airway pressure; PVI: pulmonary vein isolation.

**Table 4:** Risk of AF recurrence after catheter ablation.

Investigator	Number of patients	Ablation strategy	Diagnosis of OSA	Results: recurrence of AF in patients with OSA versus no OSA [86]
Patel et al. (2010) [78]	3000	PVI plus left atrial linear ablation	PSG	RR 1.23 [1.06,1.43]
Matiello et al. (2010) [87]	174	PVI plus left atrial linear ablation	PSG	RR 1.53 [1.21,1.92]
Chilukuri et al. (2010) [88]	109	PVI	BQ	RR 0.97 [0.75,1.26]
Tang et al. (2009) [89]	178	PVI	BQ	RR 1.03 [0.61,1.73]
Chilukuri et al. (2009) [90]	210	PVI	BQ	RR 1.18 [0.93,1.49]
Jongnarangsin et al. (2008) [91]	324	PVI plus CAFE ablation	PSG	RR 1.61 [1.16,2.22]

PVI: pulmonary vein isolation; PSG: polysomnogram; RR: risk ratio; BQ: Berlin Questionnaire; CAFE: complex fractionated atrial electrograms.

Adapted from Ng et al. 2011 [86].

**Table 5:** OSA and stroke risk.

Investigator	Number of patients	Primary endpoints	Mean followup (yrs)	Control for AF	Risk factor adjustments	Results for stroke
Boden-Albala et al. (2012) [92]	2088	Ischemic stroke, MI, death	5.1	Yes	Age, sex, race, education, WC, systolic and diastolic BP, fasting glucose, HDL, alcohol, smoking, physical activity, PVD, CAD, total cholesterol/HDL level, depression, and medication usage.	HR for ischemic stroke in patients with significant dozing was 2.74 (1.38–5.43); HR for all stroke, 3.00 (1.57–5.73)
Redline et al. (2010) [93]	5422	Ischemic stroke	8.7	No	Age, race, BMI, smoking, BP, antihypertensives, DM.	Adjusted HR for stroke in men with severe OSA was 2.86 (1.10–7.39)
Munoz et al. (2006) [94]	394	Stroke	4.5	Yes	Sex.	Adjusted HR for stroke in OSA patients was 2.52 (1.04–6.10)
Yaggi et al. (2005) [95]	1022	Stroke or death	3.4	Yes	Age, race, sex, smoking, alcohol consumption, BMI, AF, HTN, and lipids.	Unadjusted RR for stroke in OSA was 3.02 (1.27–7.21), death adjusted RR, 1.70 (0.92–3.16)
Arzt et al. (2005) [45]	1189	Stroke	4	No	Age, sex, and BMI.	Fully adjusted OR for stroke in severe OSA was 3.08 (0.74–12.81)
Marin et al. (2005) [96]	1010	Fatal MI, stroke	10.1	No	Age, CV disease, DM, HTN, lipid disorders, smoking, alcohol use, BP, glucose, lipid levels, and CV drugs.	OR for fatal MI or stroke in untreated severe OSA was 2.87 (1.17–7.51)
Mooe et al. (2001) [97]	408	Death, cerebrovascular events, MI	5.1	No	Age, sex, BMI, HTN, DM, left ventricular function, and coronary intervention.	OR for stroke in moderate OSA patients was 2.62 (1.26–5.46), in severe OSA patients, 2.98 (1.43–6.20)
Hu et al. (2000) [98]	71,779 women	Stroke, coronary heart disease, fatal cardiovascular events	8	No	Age, BMI, cigarette smoking, DM, hypercholesterolemia, menopausal status, family history of MI before age 60, alcohol consumption, multivitamin and vitamin E use, physical activity, number of hours sleeping, and sleep position.	Age-adjusted total stroke RR for occasional snorers, 1.60 (1.21–2.12); for regular snorers 1.88 (1.29–2.74). Multivariate adjusted RR for occasional snorers, 1.42 (1.07–1.89); for regular snorers RR 1.35 (0.91–1.99)

MI: myocardial infarction; WC: waist circumference; BP: blood pressure; HDL: high density lipoprotein; PVD: peripheral vascular disease; CAD: coronary artery disease; BMI: body mass index; HR: hazard ratio; DM: diabetes mellitus; HTN: hypertension; RR: relative risk; OR: odds ratio; CV: cardiovascular.
